# Retinal ganglion cell degeneration in glaucoma disrupts HPA axis temporal organization and dampens corticosterone production

**DOI:** 10.1111/jne.70182

**Published:** 2026-04-24

**Authors:** Pietra Souza Barsanele, Juliano Jefferson da Silva, Bryan Fellipe da Silva Cortes, Eliz Maria de Oliveira Furtado, José Cipolla‐Neto, Leonardo Vinícius Monteiro de Assis, Maristela Oliveira Poletini, Maria Nathália Moraes

**Affiliations:** ^1^ Laboratório de Cronobiologia Molecular, Departamento de Ciências Biológicas Instituto de Ciências Ambientais Químicas e Farmacêuticas, Universidade Federal de São Paulo Diadema Brazil; ^2^ Programa de Pós‐graduação em Fisiologia Instituto de Biociências, Universidade de São Paulo São Paulo Brazil; ^3^ Laboratório de Neurobiologia, Departamento de Fisiologia e Biofísica Instituto de Ciências Biomédicas, Universidade de São Paulo São Paulo Brazil; ^4^ Department of Chemistry and Molecular Biology University of Gothenburg Gothenburg Sweden; ^5^ Wallenberg Centre for Molecular and Translational Medicine, University of Gothenburg Gothenburg Sweden; ^6^ Institute of Neurobiology, Center of Brain Behavior & Metabolism, University of Lübeck Lübeck Germany; ^7^ University Hospital Schleswig‐Holstein, Campus Lübeck Lübeck Germany; ^8^ Departamento de Fisiologia e Biofísica Instituto de Ciências Biológicas, Universidade Federal de Minas Gerais Belo Horizonte Brazil

**Keywords:** circadian rhythm, neuroendocrine regulation, retinal degeneration

## Abstract

Glaucoma is a chronic optic neuropathy characterized by progressive vision loss. A previous study from our group showed that glaucoma‐induced retinal degeneration disrupts photic signaling to the suprachiasmatic nucleus (SCN), altering the molecular components of the central circadian clock. Through its hypothalamic projections, the SCN entrains the hypothalamic–pituitary–adrenal (HPA) axis and drives the rhythmic secretion of corticosterone. In this study, we investigated whether central circadian clock disruption in glaucoma impacts the HPA axis and its downstream physiological rhythms. We analyzed the temporal profiles of key genes controlling the HPA axis in mice with glaucoma. The *Crh* gene expression was reduced in the paraventricular nucleus, while *Crh‐r1* exhibited a 10‐h phase delay in the pituitary in response to glaucoma. Additionally, *Pomc* in the pituitary and *Mc2r* in the adrenal lost rhythmicity. The modulation of the daily rhythms of these key genes was associated with alterations in the diurnal rhythms of clock genes in the PVN, pituitary and adrenal gland. Glaucoma‐induced phase shifts and amplitude alterations in the rhythmic expression of *Per1*, *Per2*, *Nr1d1*, and *Bmal1* in the pituitary and adrenal gland, resulted in a temporal misalignment between the pituitary and adrenal rhythms. These molecular changes were associated with reduced corticosterone amplitude, suggesting impaired communication between central and peripheral clocks. Together, these findings demonstrate that glaucoma alters the temporal coordination of the HPA axis, highlighting how retinal dysfunction can propagate beyond the visual system to disturb systemic circadian and neuroendocrine regulation.

## 1 INTRODUCTION

The circadian timing system coordinates multiple physiological and behavioral processes, enabling organisms to adapt to daily environmental fluctuations.^1^, ^2^ Glucocorticoids (GCs) are adrenal steroid hormones that play a crucial role in adaptive responses to various types of stress and are under the control of the hypothalamus–pituitary–adrenal (HPA) neuroendocrine axis.^3^, ^4^ Beyond their well‐known rise during stress responses, GC levels also exhibit a strong circadian rhythm, peaking at the beginning of the organism's active phase, even under relatively stable, non‐stressful conditions.^5^, ^6^ The GC's circadian rhythm ensures metabolic adjustments for supplying energy before the onset of arousal in both humans and rodents. Its dysregulation has been associated with diverse pathological conditions by disrupting carbohydrate and lipid metabolism, the immune response, cardiovascular function, mood, and cognitive/brain function.^7^

In mammals, the suprachiasmatic nucleus (SCN) located in the hypothalamus is the central circadian clock, orchestrating peripheral oscillators and regulating endocrine, metabolic, and behavioral functions.^8^, ^9^ The SCN is synchronized to the light–dark (LD) cycle via a subset of intrinsically photosensitive retinal ganglion cells (ipRGCs), which convey photic information from the retina to the central clock.^10^, ^11^, ^12^ This pathway is critical for the entrainment of behavioral circadian rhythms and endocrine responses, including the rhythmic production and secretion of GCs.^5^, ^13^, ^14^ Thus, the integrity of ipRGCs is essential for maintaining communication between environmental cues and hormonal rhythmicity.^15^, ^16^

Once synchronized to environmental cues, the SCN conveys temporal signals through vasopressin (AVP) producing neurons that modulate the hypothalamic components of the HPA axis.^17^, ^18^, ^19^ In this axis, corticotropin‐releasing hormone (CRH) and AVP secreted by paraventricular nucleus (PVN) neurons stimulate the anterior pituitary. CRH binds to CRH‐R1 on anterior pituitary corticotrophs, promoting *Pomc* gene transcription and the post‐translational processing of the POMC prohormone.^20^, ^21^, ^22^ After POMC cleavage, the adrenocorticoid hormone (ACTH) rises in circulation, and it activates adrenal steroidogenesis through melanocortin 2 receptor (MCR2), driving GC release.^23^, ^24^, ^25^ In addition, SCN regulates autonomic inputs to the adrenal gland through innervation to the autonomic PVN neurons.^26^ This latter regulation is particularly relevant to the corticosterone release in response to light, which may contribute to the photoentrainment of CG circadian rhythm.^27^

The molecular mechanism involving clock genes is another layer of circadian regulation of the HPA activity,^28^ which is expressed in both central biological clock and across peripheral tissues.^3^, ^4^, ^29^ Protein such as BMAL1, PER1/2, CRY1/2, REV‐ERB‐a, ROR and CLOCK drive transcriptional‐translational feedback loops, generating circadian oscillations in gene expression of approximately 24 h.^2^, ^30^ The CLOCK: BMAL‐1 transcriptional factor regulates the StAR, the limiting enzyme of steroidogenesis.^31^ In the other way around, the clock genes *Per1* and *Per2* possess elements responsive to GCs (GREs) in their promoter region, indicating reciprocal regulation between CGs and the circadian clock.^32^, ^33^

Glaucoma, a progressive optic neuropathy associated with elevated intraocular pressure (IOP), leads to the degeneration of RGCs and disrupts SCN‐mediated circadian synchronization.^34^, ^35^, ^36^, ^37^ We have previously demonstrated that elevated IOP in experimental models of glaucoma alters the molecular clock components of the SCN, thereby disrupting the synchronization of circadian‐regulated behaviors such as locomotor activity and body temperature in mice.^37^ Patients with primary open‐angle glaucoma (POAG) display a phase delay and reduced amplitude in the circadian rhythm of body temperature, accompanied by sleep disturbances, such as delayed sleep onset and shorter sleep duration.^38^, ^39^, ^40^, ^41^ These patients also exhibit circadian hormonal dysregulation shaped by seasonal modulation, resulting in altered salivary melatonin and cortisol profiles.^42^ Moreover, patients with severe glaucoma also show lower levels of melatonin metabolites in the first morning urine, supporting an impairment of circadian‐regulated hormone secretion.^43^

The specific molecular components of the HPA axis affected in glaucoma, in response to RGC loss, remain unclear. Here, we assessed the hypothesis that advanced glaucoma disrupts the transcriptional regulation of key molecular components of the HPA axis, leading to impaired circadian control of its downstream physiological processes. We found reduced transcript levels of *Avp*, *Crh*, and *Nr3c1* in the PVN at the light phase, accompanied by a phase delay in *Crh‐r1* expression in the pituitary, and a loss of temporal oscillation of *Pomc* in the pituitary and *Mc2r* in the adrenal gland. Advanced glaucoma was associated with disrupted rhythmic transcript of clock genes in the PVN, pituitary and adrenal gland. Consistent with these molecular changes, plasma corticosterone levels were reduced throughout the diurnal cycle, highlighting that glaucoma‐associated RGC degeneration impairs circadian regulation of the HPA axis.

## 2 MATERIALS AND METHODS

### 2.1 Mouse strain and housing condition

We employed the DBA/2 J mouse strain as a well‐established model of spontaneous glaucoma. This strain carries a mutation in the *Gpnmb* (*glycoprotein non‐metastatic melanoma protein B*) gene, which results in elevated IOP and progressive retinal degeneration.^37^, ^44^, ^45^ Age‐matched DBA/2J‐*Gpnmb* ^+^ (SjJ) mice, which do not carry the *Gpnmb* mutation and therefore do not develop glaucomatous pathology, were used as controls (Jackson Laboratory, RRID: IMSR_JAX:007048 and IMSR_JAX:000671, respectively). All experimental procedures were conducted in accordance with national and international ethical guidelines and approved by the Institutional Animal Care and Use Committees of ICB/USP (8143290819) and ICAQF/UNIFESP (2151020922). Male mice were group‐housed (2–5 per cage) at 25 ± 1°C in a 12 h light/12 h dark (LD 12:12) cycle. Lighting was provided by LED lamps at 400 lux (420–750 nm). Food and water were available *ad libitum* throughout the study. The lights were turned on at 7 AM (zeitgeber time—ZT0) and turned off at 7 PM (ZT12).

### 2.2 RNA extraction, rt‐PCR and real time PCR

Control (*n* = 43) and glaucomatous (*n* = 37) mice at 12‐month‐old were euthanized every 4 h from ZT0 (lights on) for circadian gene expression analysis in the pituitary and adrenal glands. For the PVN, animals (13 control and 12 glaucomatous) were euthanized at two‐time points, ZT4 (four hours after lights on) and ZT16 (four hours after lights off). Coronal hypothalamic sections (800 μm) were obtained using a cryostat, using the Paxinos atlas as a reference for the region encompassing the PVN (bregma −0.46 to −1.22 mm). From this section, a microdissection using a 1‐mm diameter punch needle was obtained. Total RNA extraction from each tissue was performed using TRIzol reagent, according to the manufacturer's instructions (Thermo Fisher Scientific, Waltham, MA, USA). For the cDNA synthesis, 1 μg of total RNA was used in a reaction with the Superscript III Reverse Transcriptase, random hexamer primers (100 ng) and RNAse out according to the manufacturer's instructions (Thermo Fisher Scientific, Waltham, MA, USA). Gene expression was quantified by real‐time PCR using 10 ng of cDNA from the PVN and 5 ng from both the pituitary and adrenal gland, on a QuantStudio 5 Flex Real‐Time PCR System (Applied Biosystems, RRID:SCR_020240) with SYBR Green chemistry (Applied Biosystems). The primer sequences were designed to span exon‐exon junctions and synthesized by IDT (Coralville, IA, USA) (Table 1). To normalize target gene expression, *Rpl19* was used as the endogenous control for the pituitary, while *Rpl37a* was used for the PVN and adrenal gland. The final concentration of each primer was 300 nM.

**TABLE 1:** Gene access numbers and sequences of primers.

Gene (access number)	Primers
*Avp* (NM_009732.2)	Forward: 5′‐CGAGTGCCACGACGGTTTTT‐3′
	Reverse: 5′‐CAGAATCCACGGACTCCCG‐3′
*Bmal1* (NM_001243048)	Forward: 5′‐AAGCTTCTGCACAATCCACAGCAC‐3′
	Reverse: 5′‐TGTCTGGCTCATTGTCTTCGTCCA‐3′
*Crh* (NM_205769.3)	Forward: 5′‐CAACCTCAGCCGGTTCTGAT‐3′
	Reverse: 5′‐GGAAAAAGTTAGCCGCAGCC‐3′
*Crh‐r1* (NM_007762.5)	Forward: 5′‐CCGCTACAACACCACAAACAAT‐3′
	Reverse: 5′‐CGCAGGATGAAAGCCGAGAT‐3′
*Mcr‐2* (NM_001271717.1)	Forward: 5′‐CCGCACCATCATCACCCTAA‐3′
	Reverse: 5′‐AGGGAACAGCGATGTGAAGG‐3′
*Nr3c1* (NM_008173.4)	Forward: 5′‐GTCGAAGGACAGCACAATTACC‐3′
	Reverse: 5′‐CGGCATGCTGGACAGTTTTT‐3′
*Pcsk1* (NM_013628.3)	Forward: 5′‐CACAGACCAGCGAATAACAAGC‐3′
	Reverse: 5′‐ATCAGCCAAATCCACCAGAGC‐3′
*Per1* (NM_0011065.3)	Forward: 5′‐AGCAGGTTCAGGCTAACCAGGAAT‐3′
	Reverse: 5′‐AGGTGTCCTGGTTTCGAAGTGTGT‐3′
*Per2* (NM_011066.3)	Forward: 5′‐ACCCTGAAAAGGAAGTGCGA‐3′
	Reverse: 5′‐GCCATATCTTCTACCGTCTCTAGC‐3′
*Pomc* (NM_001278582.1)	Forward: 5′‐CTCCAATCTTGTTTGCCTCTGC‐3′
	Reverse: 5′‐TCCAGCGAGAGGTCGAGTTT‐3′
*Star* (NM_011485.5)	Forward: 5′‐CGCTACGTTCAAGCTGTGTG‐3′
	Reverse: 5′‐TCCAGTTGAGAACCAAGCAGA‐3′
*Rpl17α* (NM_009084.4)	Forward: 5′‐CGGCGACATGGCTAAACG‐3′
	Reverse: 5′‐ACGGCTCGTCTCTTCATCTTG‐3′
*Rpl19* (NM_009078.2)	Forward: 5′‐GAAATCGCCAATGCCAACTC‐3′
	Reverse: 5′‐CAGTACCCTTCCTCTTCCCTATG‐3′
*Nr1d1* (NM_145434.4)	Forward: 5′‐AAGACATGACGACCCTGGAC‐3′
	Reverse: 5′‐CCATGCCATTCAGCTTGGTAAT‐3′

The gene expression assays were quantified according to the 2‐^ΔΔCT^ method as described by Livack.^46^ The ΔCt values were calculated by subtracting the Ct value of the reference gene (*Rpl19* in the pituitary and *Rpl37a* in the PVN and adrenal) from the Ct value of the target gene. The threshold cycle (CT) values were normalized to the reference genes (*Rpl19* in the pituitary and *Rpl37a* in the PVN and adrenal) to obtain ΔCT. The ΔΔCt values were obtained by normalizing each experimental sample to the control group (minimal mean). The ΔCT of each experimental sample was compared to the highest mean ΔCT value of the control group, resulting in ΔΔCT. Finally, relative gene expression levels were calculated using a negative exponential of base 2 (2‐^ΔΔCT^).

### 2.3 Corticosterone concentration

Trunk blood samples were obtained every 4 h from ZT0 (lights on) in 12‐month‐old control and glaucomatous mice maintained under LD cycle (*n* = 5–7 per time point). The corticosterone levels were measured in plasma diluted 1:100 in the assay buffer and analyzed in duplicate in 96‐well plates following the manufacturer's instructions for the DetectX Corticosterone Immunoassay Kit (Arbor Assays, Ann Arbor, MI, USA). The absorbance was read at 450 nm, and corticosterone concentrations were calculated from the standard curve using a four‐parameter logistic (4PL) fit via the manufacturer's online calculator and expressed as pg/mL.

### 2.4 Statistical analysis

Normality was assessed using the Shapiro–Wilk test and potential outliers were identified using the ROUT method (Q = 1%) in GraphPad Prism. Differences in PVN, pituitary and adrenal gland logarithmic data, and corticosterone levels were assessed using two‐way ANOVA followed by the Bonferroni post‐test. Rhythmic parameters of gene expression in the pituitary and adrenal glands, and plasma corticosterone levels were analyzed using the CircaCompare algorithm.^47^ CircaCompare fits a joint nonlinear cosinor model simultaneously to data from both groups, incorporating difference parameters that directly estimate and test between‐group differences in estimate acrophase (the time at which the fitted cosine curve reaches its maximum), MESOR (Midline Estimating Statistic of Rhythm), and amplitude (half the peak‐to‐trough difference). Differences in each rhythmic parameter were assessed by testing whether the corresponding difference parameter differed significantly from zero, based on t‐statistics derived from the least nonlinear squares fit. Rhythmicity for each group was confirmed prior to phase comparison by testing whether the estimated amplitude differed significantly from zero (*p* < .05); MESOR and amplitude comparisons were performed irrespective of rhythmicity status. Adjusted curves were generated using the Cosinor online tool (https://cosinor.online/appNew/index.php). All statistical analyses and graphs were carried out in GraphPad Prism 9.0 (San Diego, CA, USA, RRID:SCR_002798). The value of *p* < .05 was considered statistically significant.

### 2.5 Artificial Intelligence Statement

The authors used ChatGPT and Grammarly to improve readability and language, then reviewed and edited the content, taking full responsibility for the publication content.

## 3 RESULTS

**FIGURE 1:**
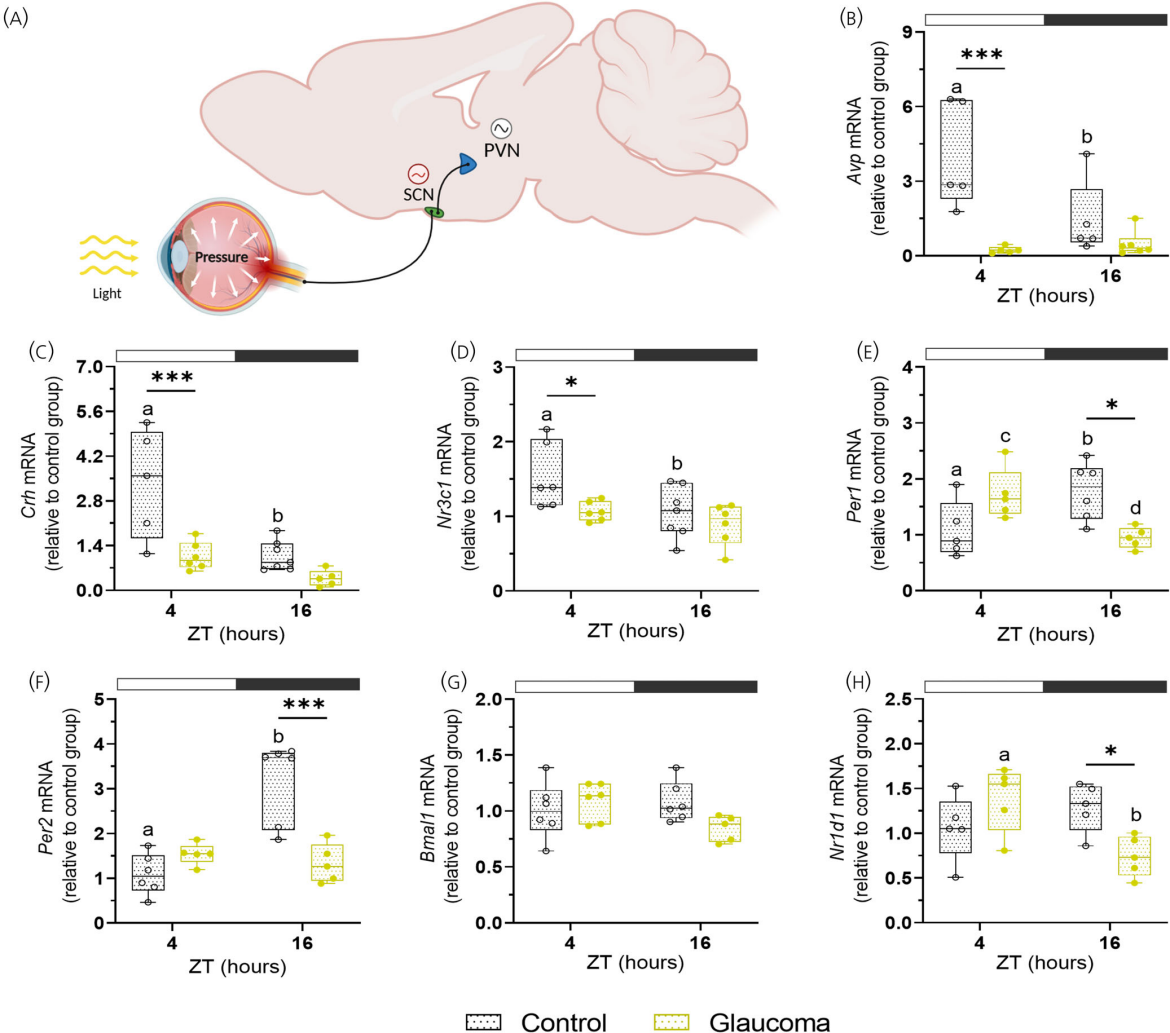
Light–dark differences in *Avp, Crh, Nr3c1* and clock gene expression in the PVN of mice with glaucoma. ZT4 lights on, ZT16 lights off. (A) Schematic representation of the retinal input to the SCN and its projection to the PVN. (B−H) Gene expression in the PVN of control and glaucomatous mice. Gene expression was analyzed using the 2 − ΔΔCT method (*n* = 5–7 for the control group and *n* = 5–6 for the glaucoma group). Boxplots show the median, quartiles, maximum, and minimum values. Symbols (circle or square) indicate the sample size for the gene of interest normalized by *Rpl37a* and expressed relative to the lowest mean of the control group. Asterisk (*) indicates a significant difference (p < 0.05) between control and glaucomatous mice, calculated by two‐way ANOVA followed by Bonferroni post‐test. The white and dark gray rectangular shapes above the graph indicate the time points corresponding to the light and dark phases, respectively. Schematic representation in (A) was created in BioRender. Barsanele, P. (2026) https://biorender.com/avtbev3.

Through direct efferent projections, partly mediated by AVPergic neurons, the SCN synchronizes the PVN, coordinating its molecular clock and regulating the temporal activity of the HPA axis.^26^, ^48^, ^49^, ^50^ To investigate the role of SCN–PVN communication in HPA axis regulation, we assessed diurnal molecular regulation within the HPA axis and measured plasma corticosterone concentrations. We found reduced *Avp* expression at ZT4 (*p* = .0005; Figure 1B) in PVN of mice with glaucoma. In controls, *Avp* expression was higher in the light phase than in the dark phase (*p* = .0124), whereas this difference was not observed in the glaucoma group. AVP released from SCN projections reaches the PVN, where vasopressinergic input is thought to contribute to the coordination of local circuits involved in the circadian regulation of HPA‐axis activity.^50^, ^51^ Control animals exhibited a significant light–dark difference (*p* = .0005) in *Crh* mRNA, with peak expression at ZT4, whereas glaucomatous mice displayed lower *Crh* expression at ZT4 compared to control (*p* = .0007) and no temporal variation (Figure 1C). We examined the GC receptor gene (*Nr3c1*, known as *GR*), which mediates corticosterone negative feedback in the PVN and is essential for maintaining rhythmicity of the HPA axis.^24^, ^51^ Upon corticosterone binding, NR3C1 regulates the expression of both clock‐controlled and stress‐related genes, thus coordinating corticosterone secretion with molecular timing mechanisms.^52^, ^53^ We found that *Nr3c1* expression follows a similar pattern to the previously analyzed genes: glaucomatous mice exhibited a significant reduction in mRNA levels during the light phase (ZT4) compared to controls (*p* = .0265; Figure 1D), whereas control mice showed a significant light–dark difference with a peak expression at the light phase (*p* = .0386).

We also analyzed the expression of molecular clock components in the PVN of glaucomatous mice. We observed a significant reduction in *Per1* mRNA (*p* = .0125) and *Per2* mRNA (*p* < .0001) at the dark phase (ZT16) in glaucomatous mice compared to controls (Figure 1E,F). *Per1* expression showed a significant day–night difference in both groups (control: *p* = .0366; glaucoma: *p* = .0256), suggesting that the temporal regulation is preserved. Notably, the peak expression of *Per1* occurred at ZT16 in control and at ZT4 in glaucomatous mice, suggesting a potential phase shift in rhythmic transcription, although formal evaluation of the diurnal profile was limited by sampling at only two‐time points. In contrast, *Per2* transcription lost its day‐night variation in glaucomatous mice (*p* = .0697), whereas the control group showed high expression at ZT16 compared to ZT4 (*p* < .001; Figure 1F). No statistical differences in *Bmal1* transcription were observed between groups or ZTs (Figure 1G). *Nr1d1* transcription (encoding REV‐ERBα) was reduced in glaucomatous mice during the dark phase compared with controls (*p* = .0283; Figure 1H). While control mice showed no differences between time points, glaucomatous mice displayed higher expression at ZT4 than at ZT16 (*p* = .0100). Overall, glaucomatous mice exhibited a temporal pattern of gene dysregulation: HPA axis–related genes were downregulated during the light phase, whereas molecular clock components showed greater reductions during the dark phase (Table S1). However, it is important to consider that PVN samples comprise multiple neuronal populations and do not allow discrimination between magnocellular and parvocellular subtypes, which may differentially contribute to the observed transcriptional changes.

**FIGURE 2:**
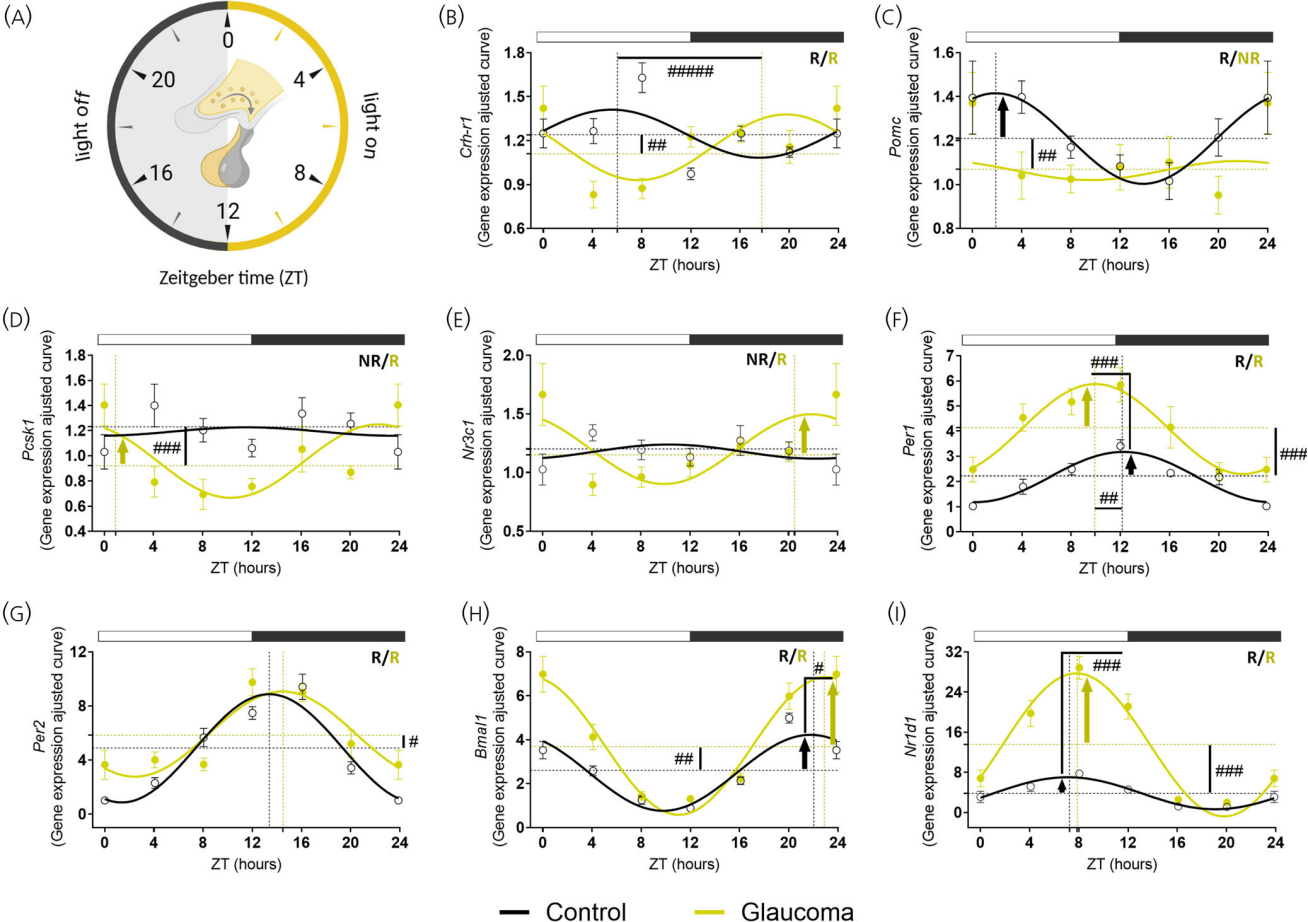
Diurnal rhythms of neuropeptides are altered in the pituitary gland of glaucomatous mice. (A) Schematic clock illustrating the analyzed zeitgeber times. Light phase (ZT0, 4, 8) and dark phase (ZT12, 16, 20), with ZT24 shown as a double plot of ZT0. (B–I) Rhythmic profiles of gene expression in the pituitary of control and glaucomatous mice. Gene expression was analyzed using the 2^‐ΔΔCT method (*n* = 4–7 for both control and glaucoma groups). Symbols represent the sample mean for the genes of interest, normalized to *Rpl19* and expressed relative to the lowest mean of the control group. The symbol # indicates p < 0.05 for rhythmic parameters between control and glaucoma groups, calculated using the CircaCompare algorithm. Cosinor Online–fitted curves show acrophase (dashed vertical line), mesor (dashed horizontal line), and amplitude (vertical arrow) as calculated by CircaCompare. White and dark gray rectangles above the graph indicate light and dark phase time points, respectively. R, rhythmic; NR, non‐rhythmic. Schematic representation in (A) was created in BioRender. Barsanele, P. (2026) https://biorender.com/2pu1xvc.

We examined how the alterations in the PVN could affect the transcriptional regulation in the pituitary (Table S2). Once released from PVN neurons, CRH reaches the anterior pituitary, where it binds to CRH‐R1 receptors, triggering a signaling cascade that stimulates ACTH production and release.^54^, ^55^ Here, we found that *Crh‐r1* exhibited rhythmic expression in both groups in the pituitary (Figure 2B). Although *Crh‐r1* rhythmicity was preserved between groups, glaucomatous mice exhibited reduced mesor (*p* = 0.0305) without changes in amplitude. A phase delay of 10 h shifted the peak expression of *Crh‐r1* from the beginning of the light phase (ZT 6:02) to the dark phase (ZT 17:52) in glaucomatous mice (Figure 2B, Table S3). Upon binding to its receptor, CRH induces transcription of *Pomc*, the precursor of ACTH, which in turn regulates corticosterone secretion in the adrenal gland.^5^, ^56^ Analysis of *Pomc* expression showed a diurnal variation only in the control group, with higher transcription at the beginning of the light phase (Figure 2C). The glaucoma group exhibited significant reductions in mesor (*p* = .0131) and amplitude (*p* = .0300), which was associated with the absence of a diurnal rhythm (Table S3). Next, we evaluate the transcription of the enzymes responsible for processing POMC into ACTH, a cleavage process conducted by PCSK1. While *Pcsk1* expression was arrhythmic in control mice (Figure 2D) glaucomatous mice showed a significant reduction in mesor (*p* = .0002) (Table S3). Consistent with this, *Nr3c1* expression in the pituitary was arrhythmic in control mice (*p* = .5739), but a pronounced diurnal rhythm in the glaucoma group was observed (*p* = .0081, Figure 2E), peaking *Nr3c1* expression at the end of the dark phase (ZT20, *p* = .0367, Figure 2E).

Considering the diurnal gene modulation in the pituitary of glaucomatous mice, we investigated whether these results were associated with changes in clock gene expression (Figure 2F–I, Table S2). While all clock genes maintained rhythmic expression in glaucomatous mice, the modulation of rhythmic parameters occurred in a gene‐specific manner. For instance, *Per1* expression exhibited a clear oscillatory profile in both groups (Figure 2F). Glaucoma mice exhibited higher *Per1* amplitude (*p* = .0228) and mesor (*p* < .0001) and an approximately 2‐h phase advance in the peak expression (ZT10) compared to controls (ZT12, Table S3). *Per2* rhythmicity was also present in both groups, with only a slight increase in the mesor in glaucomatous mice (Figure 2G). In line with the *Pers* genes, *Bmal1* expression displayed significant temporal variation in both groups (Figure 2H). The glaucoma group showed an increased *Bmal1* amplitude (*p* = .0011), and mesor (*p* < .0001) compared with controls (Table S3). Furthermore, *Nr1d1 expression*, which was rhythmic in both groups (Figure 2I), displayed a significantly higher amplitude and mesor in glaucomatous mice relative to controls (Table S3).

**FIGURE 3:**
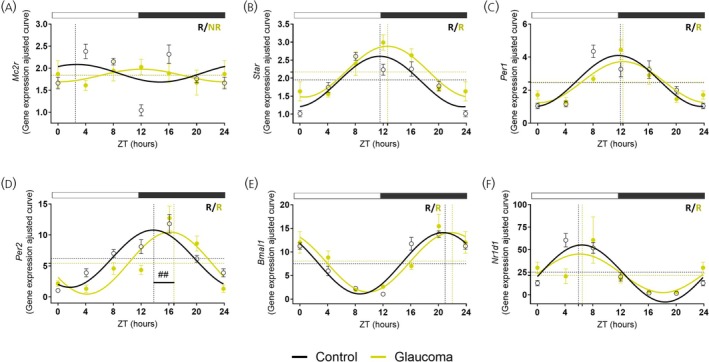
Glaucoma mainly affects adrenal *Mc2r* circadian profile. Light phase (ZT0, 4, 8) and dark phase (ZT12, 16, 20), with ZT24 shown as a double plot of ZT0. (A–F) Rhythmic profiles of gene expression in the adrenal gland of control and glaucomatous mice. Gene expression was analyzed using the 2^‐ΔΔCT method (*n* = 4–8 for the control group and *n* = 3–7 for the glaucoma group). Symbols represent the sample mean for the genes of interest, normalized to *Rpl37a* and expressed relative to the lowest mean of the control group. The symbol # indicates *p* < .05 for rhythmic parameters between control and glaucoma groups, calculated using the CircaCompare algorithm. Cosinor Online‐fitted curves show acrophase (dashed vertical line), mesor (dashed horizontal line), and amplitude (vertical arrow) as calculated by CircaCompare. White and dark gray rectangles above the graph indicate light and dark phase time points, respectively. R, rhythmic; NR, non‐rhythmic.

As previously mentioned, ACTH released from the pituitary gland regulates GCs by binding to MC2R in the adrenal cortex.^5^, ^24^ The temporal expression of *Mc2r* in the adrenal gland, observed in the control mice, was lost in the glaucoma group (Figure 3A, Tables S4 and S5). However, mesor and amplitude remained unchanged, suggesting a modest effect on overall gene expression. On the other hand, the diurnal rhythm of *StAR* mRNA was present in both groups with no differences in the rhythmic parameters analyzed (Figure 3B, Table S5). Temporal expression analysis of key clock genes in the adrenal revealed that *Per1*, *Bmal1*, *Per2*, and *Nr1d1* maintained robust diurnal oscillations in both control and glaucomatous mice (Figure 3C–F). No significant differences were observed in the diurnal parameters of *Per1*, *Bmal1*, or *Nr1d1* (Figure 3, Table S5). In contrast, the peak expression of *Per2* was delayed by approximately 3‐h in the glaucoma group, shifting from ZT13.83 in controls to ZT16.73 in glaucoma (Figure 3D, Table S5).

**FIGURE 4:**
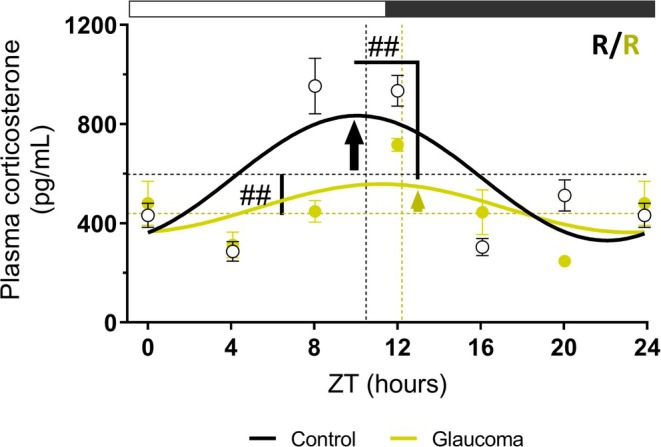
Glaucoma is associated with decreased amplitude of the circadian plasma corticosterone profile. Light phase (ZT0, 4, 8) and dark phase (ZT12, 16, 20), with ZT24 shown as a double plot of ZT0. Rhythmic profiles of plasma corticosterone in control and glaucomatous (*n* = 4–7 for the control group and *n* = 3–7 for the glaucoma group). The symbol # indicates *p* < .05 for rhythmic parameters between groups, calculated using the CircaCompare algorithm. Cosinor Online‐fitted curves show acrophase (dashed vertical line), mesor (dashed horizontal line), and amplitude (vertical arrow) as calculated by CircaCompare. White and dark gray rectangles above the graph indicate light and dark phase time points, respectively. NR, non‐rhythmic; R, rhythmic.

To evaluate whether the described transcriptional changes lead to physiological alterations in the adrenal gland, we measured plasma corticosterone levels. In control mice, corticosterone displayed a robust diurnal rhythm, with higher levels peaking before the onset of the active phase, as expected (Figure 4). Although both groups preserved an oscillatory profile, glaucoma mice exhibited a significantly reduced amplitude accompanied by a lower mesor compared to controls (Table S5).

## 4 DISCUSSION

In this study, we found that glaucoma‐induced retinal degeneration reduces corticosterone secretion by altering the temporal organization of the HPA axis. Using a murine model of late‐stage glaucoma, we identified molecular alterations that provide insights into disturbances in neuroendocrine rhythmicity observed in glaucoma patients.^42^ Previously, our group revealed that retinal neurodegeneration induced by advanced glaucoma in mice alters the molecular mechanisms involving clock genes in the SCN and affects the transcription of key neuropeptides such as *Vip* and *Avp*.^37^ Despite these rhythmic alterations, glaucoma mice remain able to entrain to the light–dark cycle and display a locomotor activity profile similar to control mice, suggesting that the observed diurnal alterations are associated with retinal degeneration rather than a complete loss of photic entrainment.^37^ Among these neuropeptides, AVP acts as a key signaling molecule of SCN efferent neurons coordinating circadian synchronization of neuroendocrine and autonomic rhythms within specific hypothalamic targets.^57^, ^58^, ^59^ SCN‐derived AVP projections to the PVN contribute to the modulation of local clock gene transcription,^51^, ^60^, ^61^ supporting temporal coordination between the PVN and the central circadian clock.

In the SCN of mice with glaucoma, a 7‐h delay in the *Avp* peak expression was demonstrated, shifting it from the middle to the beginning of the light phase.^37^ Together with the reduced AVP expression in the PVN at ZT4 (Figure 1B), these findings raise the possibility of altered temporal relationship between the SCN and PVN. Moreover, the previously reported difference in locomotor activity onset between groups, together with the phase delay in *Avp* peak expression in the SCN,^37^ suggests that the changes observed in PVN gene expression may reflect a phase shift rather than a reduction in rhythm amplitude. Notably, the PVN punches did not allow discrimination between magnocellular and parvocellular neuronal populations. Given that *Avp* expression in the PVN is predominantly derived from magnocellular neurons,^62^ the present findings should not be interpreted as a direct modulation of hypophysiotropic AVP regulation of ACTH in the pituitary. Instead, the altered day‐night expression of *Avp*, in parallel with the changes observed in *Crh* and clock gene rhythms, likely reflects an alteration in the diurnal organization of neuropeptidergic gene expression in the PVN of mice with glaucoma.

Along with changes in *Avp* expression, *Crh* and *Nr3c1* exhibited reduced expression in the PVN at the light phase in glaucoma, whereas *Per1* and *Per2* displayed a similar decrease at the dark phase (Figure 1). Since GR is specifically expressed in neuroendocrine parvocellular neurons in the PVN,^63^, ^64^ the reduced *Nr3c1* expression observed in glaucomatous mice likely reflects alterations in CRH neurons, suggesting impaired GC negative feedback. Under physiological conditions, *Crh* mRNA levels are highest at the beginning of the light phase, gradually declining throughout this period and reaching their lowest levels at the onset of the dark phase.^51^, ^65^, ^66^ Although SCN‐derived AVP is a key signal for the circadian regulation of the HPA axis, the specific PVN neuronal populations through which these effects are mediated remain to be identified.^67^, ^68^

In contrast to previous reports in rats showing no circadian rhythm of *Avp* transcription in the PVN,^69^ we observed a significant day–night difference in *Avp* mRNA in control mice. Since only two‐time points were analyzed, these data do not demonstrate circadian rhythmicity but indicate a time‐of‐day variation. In addition to possible species‐specific differences, methodological aspects should be considered, as the available study quantified *Avp* hnRNA by in situ hybridization in the medial parvocellular PVN,^69^ reflecting acute transcriptional activity in a restricted neuronal population. In contrast, our analysis measured total *Avp* mRNA in PVN punches, providing the combined gene expression of multiple neuronal populations. The markedly reduced *Avp* and *Nr3c1* expression observed in the PVN of glaucomatous mice, together with a similarly attenuated corticosterone rhythm (Figure 4), suggests that glaucoma‐induced chronodisruption impairs central diurnal signaling and affects HPA axis regulation.

The signaling cascade activated by CRH in the pituitary gland regulates *Per* transcription and contributes to clock synchronization.^5^, ^70^, ^71^ Decreased expression of *Per1* and *Per2* in the PVN at the dark phase indicates a potential alteration in their expected daily pattern (Figure 1E,F). Under physiological conditions, *Per1/2* expression peaks at the dark phase, therefore their current reduction suggests a possible phase‐dependent alteration of rhythmicity in glaucomatous mice. Given that PER1/2 in the PVN contributes not only to the local circadian clock, but also to the regulation of the timing and amplitude of CRH release,^51^ such changes could reflect altered neuroendocrine regulation. In our model, both *Per1* and *Per2* were reduced at the dark phase; however, only *Per1* shows temporal differences in the mRNA between the two‐time points analyzed here in response to glaucoma. Although further circadian profiling is needed, the time‐dependent modulation of *Pers* genes plays a key role in organizing downstream physiological processes. Indeed, rhythmic clock gene expressions within the PVN are essential for the proper coordination of GC secretion, as *Per2*‐knockout mice show a blunted corticosterone and consequent HPA dysregulation.^72^

Additionally, BMAL1 in the PVN is essential for the circadian expression of neuroendocrine genes,^51^, ^73^ a role underscored by the reduced daily corticosterone release in CRH‐specific BMAL1 knockout mice.^74^, ^75^ However, in our study, *Bmal1* expression showed only minor changes in the PVN, pituitary and adrenal gland, suggesting that alteration in corticosterone secretion is driven by alterations in other components of the HPA axis. Even though glaucoma did not disrupt the rhythmicity of pituitary and adrenal clock genes, their expression exhibited significant changes in rhythmic parameters. *Pers* genes were differentially modulated by glaucoma, with *Per1* being more affected in the pituitary and *Per2* in the adrenal gland. Typically, *Per1/2* displays tightly aligned circadian expression profiles in the pituitary and adrenal glands of nocturnal rodents, characterized by a peak at the day‐to‐night transition.^66^, ^70^ In contrast, mice with glaucoma show a phase advance of *Per1* in the pituitary and a phase delay of *Per2* in the adrenal, compromising their phase alignment.

CRH signaling pathway promotes POMC transcription in the pituitary gland, leading to ACTH release.^5^, ^76^, ^77^ The shift in *Crh‐r1* transcription from light to dark phase in glaucoma (Figure 2B), together with low *Avp* and *Crh* levels at ZT4 in the PVN (Figure 1B,C), suggests that glaucoma compromised the diurnal coordination between the PVN and the pituitary gland. Daily rhythm of *Pomc* expression in the control group aligns with previous reports.^66^ However, the loss of *Pomc* rhythmicity in glaucomatous mice may arise either from disease‐related pituitary alterations or from SCN‐driven misalignment of HPA axis timing. Since POMC is the precursor of multiple neuropeptides, its expression alone is insufficient to fully represent ACTH secretion. POMC is cleaved by PCSK1, generating pro‐ACTH and β‐LPH (beta‐lipotropin), with pro‐ACTH subsequently processed by PCSK1 to produce ACTH.^23^, ^25^ In this context, the persistence of *Pcsk1* rhythmicity when its main substrate lacks temporal oscillation could affect the efficiency or timing of ACTH processing. While the causal relationship remains unclear, such a mismatch would be consistent with the reduced adrenal stimulation and the reduced corticosterone amplitude observed in glaucoma. The loss of rhythmic *Mc2r* transcription in adrenal of glaucomatous mice (Figure 3A) further reveals that this key component of the HPA axis is also susceptible to central disruption. Even if some upstream pituitary genes are still oscillating, the absence of *Mc2r* rhythmicity may limit adrenal sensitivity to ACTH, thereby contributing to the attenuated corticosterone rhythm observed in glaucoma. Together, these alterations provide a plausible mechanistic basis for the markedly reduced amplitude of plasma corticosterone rhythm in glaucomatous mice.

In our glaucoma model, impaired light transduction as a result of RGC degeneration may partially account for the observed phase delay of the *Per2* gene in the adrenal gland, providing a potential explanation for tissue‐specific diurnal misalignment. This finding supports the notion that *Per2* transcription in the adrenal is directly regulated by photic input conveyed through the retino‐hyphothalamic tract to SCN clock and non‐clock neurons.^78^ Beyond this SCN‐mediated pathway, the retina also engages autonomic circuits, enabling temporal modulation of adrenal rhythms independently of the central circadian clock.^78^ This local rhythmic autonomy of the adrenal gland can sustain oscillatory activity even under central misalignment.^79^, ^80^, ^81^, ^82^ In fact, light exposure during darkness induces corticosterone secretion and *Per1* transcription in the adrenal without elevating plasma ACTH levels, indicating that light can drive corticosterone release independently of the HPA axis, most likely via descending autonomic circuits.^27^, ^78^, ^83^

Although autonomic pathways modulate adrenal activity, adrenal‐specific BMAL1 deletion abolishes GC rhythmicity.^79^ In addition, rhythmic *Per1*/2 and *Bmal1* expression persists in the adrenal glands of hypophysectomized rats,^84^ confirming that local adrenal clocks are essential for sustained corticosterone oscillations. The local clock may account for the maintenance of corticosterone oscillation in the glaucoma mice while the HPA axis inputs for the magnitude of the diurnal peak. Because of a reduced corticosterone secretion, the feedback regulation exerted on the axis may have been compromised. In fact, the *Nrc31* expression, which mediates corticosterone negative feedback in the PVN, was reduced in the PVN. The reduced circadian amplitude of corticosterone may also affect the SCN, as GR are present in SCN neurons.^85^ Importantly, GC signaling in the SCN modulates the expression of GC‐responsive genes which are primarily involved in stress responsiveness, neuronal excitability, and transcriptional regulation, rather than in the core molecular clock machinery.^85^ Thus, GC actions in the SCN may influence non‐clock‐related functions without directly altering the core circadian oscillator. Altogether, our findings suggest that the alterations observed in corticosterone rhythm may not be only due to disruption in the SCN induced by glaucoma,^37^ since neurodegeneration of RGCs contributes to changes in the adrenal clock. Nonetheless, through both HPA‐mediated endocrine cues and autonomic innervation, the SCN reinforces adrenal timing, coupling peripheral and central clock.^86^, ^87^ This responsiveness supports the idea that peripheral mechanisms can partially compensate for central misalignment observed in glaucomatous mice,^37^ maintaining local clock oscillations and corticosterone rhythmicity.

The progressive attenuation of glaucoma‐induced alterations along the SCN–PVN–peripheral tissues may reflect the hierarchical organization of the circadian system, in which downstream oscillators rely less on direct SCN signaling and are increasingly influenced by local and systemic cues.^88^, ^89^ Under impairment of SCN functions, as occurs in glaucoma, peripheral oscillators may increase the integration of non‐SCN temporal inputs, to preserve local rhythmicity despite weakened central signaling.^90^, ^91^ This reorganization may not be sufficient to prevent circadian misalignment, particularly in aged or advanced‐stage glaucoma, where circadian resilience may already be reduced.^92^, ^93^

These findings highlight that glaucoma‐induced neurodegeneration of RGCs is associated with alterations in central components of the HPA axis, supporting the idea that diurnal endocrine homeostasis is disrupted in this condition. Clinical evidence further shows that glaucoma patients exhibit disturbances in cortisol rhythms, along with impaired sleep quality.^42^, ^94^, ^95^ Furthermore, glaucoma‐induced temporal misalignment mirrors the circadian disruption observed in night‐shift workers, where altered cortisol patterns are linked to a higher risk of metabolic and cardiovascular disorders.^96^, ^97^, ^98^ Comparable systemic consequences related to circadian rhythms have also been documented in glaucoma patients, including disrupted body temperature rhythms and reduced nocturnal melatonin secretion,^41^, ^43^, ^99^ reinforcing that their impact extends beyond vision.

## 5 CONCLUSION

We demonstrate that glaucoma‐induced retinal degeneration impairs systemic diurnal regulation by altering the temporal organization of the HPA axis. The neurodegeneration of RGCs leads to altered rhythmic expression of key clock genes and neuropeptides, including *Per1/2*, *Nr1d1*, *Crh‐r1*, *Pomc*, and *Mc2r* across the axis. Although individual alterations in glaucomatous mice may be difficult to interpret, the reduced corticosterone rhythm reveals that these combined changes result in an overall impaired HPA axis response. These molecular alterations may trigger compensatory mechanisms, such as preserved adrenal rhythmicity, which may help sustain HPA axis function.

Among the limitations of this study is the restricted temporal sampling in the PVN, where gene expression was assessed at only two‐time points, preventing conclusions about diurnal rhythmicity and allowing only the detection of time‐of‐day differences. In contrast, pituitary and adrenal analyses included six time points, enabling a more robust assessment of diurnal rhythmicity. Additional limitations include the absence of sex‐specific analyses, as only male animals were used. These limit our conclusions in determining tissue‐specific temporal alterations, and in assessing potential sex differences in glaucoma‐related alterations. Our findings help explain the disrupted cortisol rhythms seen in humans with glaucoma, improving understanding of how circadian misalignment contributes to disease.

## AUTHOR CONTRIBUTIONS

*Conceptualization*: Maria Nathália Moraes and Pietra Souza Barsanele. *Data curation*: Pietra Souza Barsanele. *Formal analysis*: Pietra Souza Barsanele; Leonardo Vinícius Monteiro de Assis; and Maria Nathália Moraes. *Investigation*: Pietra Souza Barsanele; Juliano Jefferson da Silva; Eliz Maria de Oliveira Furtado; Bryan Fellipe da Silva Cortes; José Cipolla‐Neto; and Maria Nathália Moraes. *Methodology*: Pietra Souza Barsanele; Maristela Oliveira Poletini; and Maria Nathália Moraes. *Funding acquisition and project administration*: Maria Nathália Moraes. *Supervision*: Maristela Oliveira Poletini and Maria Nathália Moraes. *Visualization*: Pietra Souza Barsanele; Leonardo Vinícius Monteiro de Assis; Maristela Oliveira Poletini; and Maria Nathália Moraes. *Writing—original draft*: Pietra Souza Barsanele and Maria Nathália Moraes. *Writing—review and editing*: all authors. All authors have approved the definitive version of the manuscript and agreed to be accountable for all aspects of the study in ensuring that questions related to the accuracy or integrity of any part of the study are appropriately investigated and resolved. All people designated as authors qualify for authorship, and all those who qualify for authorship are listed.

## FUNDING INFORMATION

This work was partially supported by the São Paulo Research Foundation (FAPESP), grants 2017/26651‐9 and 2022/15729‐5 (Maria Nathália Moraes), 2019/24327‐5 (José Cipolla‐Neto), the National Council of Technological and Scientific Development (CNPq) grants 403599/2025‐3 (Maria Nathália Moraes) and 406445/2023‐0 (Maristela Oliveira Poletini and Maria Nathália Moraes). Pietra Souza Barsanele (2022/07969‐6 and 2025/04483‐3) and Eliz Maria Oliveira Furtado (2023/08461‐9) are fellow of FAPESP, Juliano Jefferson da Silva (88887.461510/2019‐00 and 88887.663829/2022‐00) and Bryan Fellipe da Silva Cortes are fellows of CAPES. Leonardo Vinícius Monteiro de Assis is supported by the Knut and Alice Wallenberg Foundation as a Wallenberg Molecular Medicine Fellow and by the German Research Foundation (Deutsche Forschungsgemeinschaft) grant 541063275—TRR 418 (B04).

## CONFLICT OF INTEREST STATEMENT

The authors declare no conflict of interest.

## ETHICS STATEMENT

The study was conducted according to the guidelines of the Declaration of Helsinki and approved by the Ethics Committee of the Institute of Biomedical Science, University of São Paulo (protocol CEUA ICB/USP, number 8143290819, October 2019), and by the Ethics Committee of the Department of Biological Sciences, Federal University of São Paulo (protocol CEUA number 2151020922, September 2022).

## Supporting information


**Table S1.** Two‐way analysis of gene expression in the paraventricular nucleus.
**Table S2.** Two‐way analysis of gene expression in the pituitary gland.
**Table S3.** Rhythmic parameters of gene expression in the pituitary gland.
**Table S4.** Two‐way analysis of gene expression in the adrenal gland.
**Table S5.** Rhythmic parameters of gene expression in the adrenal gland.

## Data Availability

The data that support the findings of this study are available from the corresponding author upon reasonable request.

## References

[1] Jagannath A , Taylor L , Wakaf Z , Vasudevan SR , Foster RG . The genetics of circadian rhythms, sleep and health. Hum Mol Genet. 2017;26(R2):R128‐R138. 28977444 10.1093/hmg/ddx240 PMC5886477

[2] Cox KH , Takahashi JS . Circadian clock genes and the transcriptional architecture of the clock mechanism. J Mol Endocrinol. 2019;63(4):R93‐R102. 31557726 10.1530/JME-19-0153 PMC6872945

[3] Oster H , Challet E , Ott V , et al. The functional and clinical significance of the 24‐hour rhythm of circulating glucocorticoids. Endocr Rev. 2017;38(1):3‐45. 27749086 10.1210/er.2015-1080 PMC5563520

[4] Focke CMB , Iremonger KJ . Rhythmicity matters: circadian and ultradian patterns of HPA axis activity. Mol Cell Endocrinol. 2020;501:110652. 31738971 10.1016/j.mce.2019.110652

[5] Kalsbeek A , van der Spek R , Lei J , Endert E , Buijs RM , Fliers E . Circadian rhythms in the hypothalamo‐pituitary‐adrenal (HPA) axis. Mol Cell Endocrinol. 2012;349(1):20‐29. 21782883 10.1016/j.mce.2011.06.042

[6] Dickmeis T , Weger BD , Weger M . The circadian clock and glucocorticoids‐‐interactions across many time scales. Mol Cell Endocrinol. 2013;380(1–2):2‐15. 23707790 10.1016/j.mce.2013.05.012

[7] Crown A , Lightman S . Why is the management of glucocorticoid deficiency still controversial: a review of the literature. Clin Endocrinol (Oxf). 2005;63(5):483‐492. 16268798 10.1111/j.1365-2265.2005.02320.x

[8] Begemann K , Rawashdeh O , Olejniczak I , et al. Endocrine regulation of circadian rhythms. Biol Timing Sleep. 2025;2:10. 10.1038/s44323-025-00024-6 PMC12912344 41775850

[9] Mendoza J . Brain circadian clocks timing the 24h rhythms of behavior. npj Biological Timing and Sleep. 2025;2:13. 41776262 10.1038/s44323-025-00030-8 PMC12912386

[10] Panda S , Sato TK , Castrucci AM , et al. Melanopsin (Opn4) requirement for normal light‐induced circadian phase shifting. Science. 2002;298(5601):2213‐2216. 12481141 10.1126/science.1076848

[11] Chen SK , Badea TC , Hattar S . Photoentrainment and pupillary light reflex are mediated by distinct populations of ipRGCs. Nature. 2011;476(7358):92‐95. 21765429 10.1038/nature10206 PMC3150726

[12] Cao D , Barrionuevo PA . The importance of intrinsically photosensitive retinal ganglion cells and implications for lighting design. J Sol State Light. 2015;2:10.

[13] Nicolaides NC , Charmandari E , Kino T , Chrousos GP . Stress‐related and circadian secretion and target tissue actions of glucocorticoids: impact on health. Front Endocrinol (Lausanne). 2017;8:70. 28503165 10.3389/fendo.2017.00070 PMC5408025

[14] Androulakis IP . Circadian rhythms and the HPA axis: a systems view. WIREs Mech Dis. 2021;13(4):e1518. 33438348 10.1002/wsbm.1518 PMC8900069

[15] Berson DM , Dunn FA , Takao M . Phototransduction by retinal ganglion cells that set the circadian clock. Science. 2002;295(5557):1070‐1073. 11834835 10.1126/science.1067262

[16] Gooley JJ , Lu J , Fischer D , Saper CB . A broad role for melanopsin in nonvisual photoreception. J Neurosci. 2003;23(18):7093‐7106. 12904470 10.1523/JNEUROSCI.23-18-07093.2003 PMC6740653

[17] Buijs RM , Kalsbeek A . Hypothalamic integration of central and peripheral clocks. Nat Rev Neurosci. 2001;2(7):521‐526. 11433377 10.1038/35081582

[18] Kalsbeek A , Fliers E , Hofman MA , Swaab DF , Buijs RM . Vasopressin and the output of the hypothalamic biological clock. J Neuroendocrinol. 2010;22:362‐372. 20088910 10.1111/j.1365-2826.2010.01956.x

[19] Yamaguchi Y , Maekawa Y , Kabashima K , et al. An intact pituitary vasopressin system is critical for building a robust circadian clock in the suprachiasmatic nucleus. Proc Natl Acad Sci USA. 2023;120(43):e2308489120. 37844254 10.1073/pnas.2308489120 PMC10614613

[20] Dautzenberg FM , Hauger RL . The CRF peptide family and their receptors: yet more partners discovered. Trends Pharmacol Sci. 2002;23(2):71‐77. 11830263 10.1016/s0165-6147(02)01946-6

[21] Smith SM , Vale WW . The role of the hypothalamic‐pituitary‐adrenal axis in neuroendocrine responses to stress. Dialogues Clin Neurosci. 2006;8(4):383‐395. 17290797 10.31887/DCNS.2006.8.4/ssmith PMC3181830

[22] Aguilera G , Liu Y . The molecular physiology of CRH neurons. Front Neuroendocrinol. 2012;33(1):67‐84. 21871477 10.1016/j.yfrne.2011.08.002 PMC4341841

[23] Raffin‐Sanson ML , de Keyzer Y , Bertagna X . Proopiomelanocortin, a polypeptide precursor with multiple functions: from physiology to pathological conditions. Eur J Endocrinol. 2003;149(2):79‐90. 12887283 10.1530/eje.0.1490079

[24] Nicolaides NC , Charmandari E , Chrousos GP , Kino T . Circadian endocrine rhythms: the hypothalamic‐pituitary‐adrenal axis and its actions. Ann N Y Acad Sci. 2014;1318:71‐80. 24890877 10.1111/nyas.12464 PMC4104011

[25] Chrétien M , Mbikay M . 60 YEARS OF POMC: from the prohormone theory to pro‐opiomelanocortin and to proprotein convertases (PCSK1 to PCSK9). J Mol Endocrinol. 2016;56(4):T49‐T62. 26762158 10.1530/JME-15-0261

[26] Buijs RM , Hermes MH , Kalsbeek A . The suprachiasmatic nucleus‐paraventricular nucleus interactions: a bridge to the neuroendocrine and autonomic nervous system. Prog Brain Res. 1998;119:365‐382. 10074800 10.1016/s0079-6123(08)61581-2

[27] Ishida A , Mutoh T , Ueyama T , et al. Light activates the adrenal gland: timing of gene expression and glucocorticoid release. Cell Metab. 2005;2(5):297‐307. 16271530 10.1016/j.cmet.2005.09.009

[28] Maywood ES , Reddy AB , Wong GK , et al. Synchronization and maintenance of timekeeping in suprachiasmatic circadian clock cells by neuropeptidergic signaling. Curr Biol. 2006;16(6):599‐605. 16546085 10.1016/j.cub.2006.02.023

[29] Neumann AM , Schmidt CX , Brockmann RM , Oster H . Circadian regulation of endocrine systems. Auton Neurosci. 2019;216:1‐8. 30598120 10.1016/j.autneu.2018.10.001

[30] Takahashi JS . Molecular components of the circadian clock in mammals. Diabetes Obes Metab. 2015;17 Suppl 1(1):6‐11. 26332962 10.1111/dom.12514 PMC4560116

[31] Stocco DM . StAR protein and the regulation of steroid hormone biosynthesis. Annu Rev Physiol. 2001;63:193‐213. 11181954 10.1146/annurev.physiol.63.1.193

[32] Balsalobre A , Brown SA , Marcacci L , et al. Resetting of circadian time in peripheral tissues by glucocorticoid signaling. Science. 2000;289(5488):2344‐2347. 11009419 10.1126/science.289.5488.2344

[33] Yamamoto T , Nakahata Y , Tanaka M , et al. Acute physical stress elevates mouse period1 mRNA expression in mouse peripheral tissues via a glucocorticoid‐responsive element. J Biol Chem. 2005;280(51):42036‐42043. 16249183 10.1074/jbc.M509600200

[34] Drouyer E , Dkhissi‐Benyahya O , Chiquet C , et al. Glaucoma alters the circadian timing system. PLoS One. 2008;3(12):e3931. 19079596 10.1371/journal.pone.0003931 PMC2592693

[35] de Zavalía N , Plano SA , Fernandez DC , et al. Effect of experimental glaucoma on the non‐image forming visual system. J Neurochem. 2011;117(5):904‐914. 21446997 10.1111/j.1471-4159.2011.07260.x

[36] Gao J , Griner EM , Liu M , Moy J , Provencio I , Liu X . Differential effects of experimental glaucoma on intrinsically photosensitive retinal ganglion cells in mice. J Comp Neurol. 2022;530(9):1494‐1506. 34958682 10.1002/cne.25293 PMC9010357

[37] Barsanele PS , de Assis LVM , da Silva JJ , et al. Glaucoma‐inducing retinal ganglion cell degeneration alters diurnal rhythm of key molecular components of the central clock and locomotor activity in mice. FASEB J. 2024;38(20):e70109. 39441606 10.1096/fj.202401105R

[38] Lanzani MF , de Zavalía N , Fontana H , Sarmiento MI , Golombek D , Rosenstein RE . Alterations of locomotor activity rhythm and sleep parameters in patients with advanced glaucoma. Chronobiol Int. 2012;29(7):911‐919. 22823874 10.3109/07420528.2012.691146

[39] Wang H , Zhang Y , Ding J , Wang N . Changes in the circadian rhythm in patients with primary glaucoma. PLoS One. 2013;8(4):e62841. 23658653 10.1371/journal.pone.0062841 PMC3639222

[40] Gracitelli CP , Duque‐Chica GL , Roizenblatt M , et al. Intrinsically photosensitive retinal ganglion cell activity is associated with decreased sleep quality in patients with glaucoma. Ophthalmology. 2015;122(6):1139‐1148. 25858174 10.1016/j.ophtha.2015.02.030

[41] Gubin DG , Malishevskaya ТN , Astakhov YS , et al. Progressive retinal ganglion cell loss in primary open‐angle glaucoma is associated with temperature circadian rhythm phase delay and compromised sleep. Chronobiol Int. 2019;36(4):564‐577. 30663431 10.1080/07420528.2019.1566741

[42] Østergaard Madsen H , Hageman I , Kolko M , Lund‐Andersen H , Martiny K , Ba‐Ali S . Seasonal variation in neurohormones, mood and sleep in patients with primary open angle glaucoma – implications of the ipRGC‐system. Chronobiol Int. 2021;38(10):1421‐1431. 34112046 10.1080/07420528.2021.1931275

[43] Yoshikawa T , Obayashi K , Miyata K , Saeki K , Ogata N . Decreased melatonin secretion in patients with glaucoma: Quantitative association with glaucoma severity in the LIGHT study. J Pineal Res. 2020;69(2):e12662. 32333450 10.1111/jpi.12662

[44] Libby RT , Anderson MG , Pang IH , et al. Inherited glaucoma in DBA/2J mice: pertinent disease features for studying the neurodegeneration. Vis Neurosci. 2005;22(5):637‐648. 16332275 10.1017/S0952523805225130

[45] Howell GR , Libby RT , Marchant JK , et al. Absence of glaucoma in DBA/2J mice homozygous for wild‐type versions of Gpnmb and Tyrp1. BMC Genet. 2007;8:45. 17608931 10.1186/1471-2156-8-45 PMC1937007

[46] Livak KJ , Schmittgen TD . Analysis of relative gene expression data using real‐time quantitative PCR and the 2(‐delta delta C(T)) method. Methods. 2001;25(4):402‐408. 11846609 10.1006/meth.2001.1262

[47] Parsons R , Parsons R , Garner N , Oster H , Rawashdeh O . CircaCompare: a method to estimate and statistically support differences in mesor, amplitude and phase, between circadian rhythms. Bioinformatics. 2020;36(4):1208‐1212. 31588519 10.1093/bioinformatics/btz730

[48] Yamase K , Takahashi S , Nomura K , Haruta K , Kawashima S . Circadian changes in arginine vasopressin level in the suprachiasmatic nuclei in the rat. Neurosci Lett. 1991;130(2):255‐258. 1795892 10.1016/0304-3940(91)90409-m

[49] Dai J , Swaab DF , Buijs RM . Distribution of vasopressin and vasoactive intestinal polypeptide (VIP) fibers in the human hypothalamus with special emphasis on suprachiasmatic nucleus efferent projections. J Comp Neurol. 1997;383(4):397‐414. 9208989 10.1002/(sici)1096-9861(19970714)383:4<397::aid-cne1>3.0.co;2-y

[50] Tousson E , Meissl H . Suprachiasmatic nuclei grafts restore the circadian rhythm in the paraventricular nucleus of the hypothalamus. J Neurosci. 2004;24(12):2983‐2988. 15044537 10.1523/JNEUROSCI.5044-03.2004 PMC6729855

[51] Jones JR , Chaturvedi S , Granados‐Fuentes D . Circadian neurons in the paraventricular nucleus entrain and sustain daily rhythms in glucocorticoids. Nat Commun. 2021;12:5763. 34599158 10.1038/s41467-021-25959-9 PMC8486846

[52] Laryea G , Muglia L , Arnett M , Muglia LJ . Dissection of glucocorticoid receptor‐mediated inhibition of the hypothalamic‐pituitary‐adrenal axis by gene targeting in mice. Front Neuroendocrinol. 2015;36:150‐164. 25256348 10.1016/j.yfrne.2014.09.002 PMC4342273

[53] Lee H , Shams S , Dang Thi VH , et al. Key HPI axis receptors facilitate light adaptive behavior in larval zebrafish. Res Sq. 2024;14(1):7759. 10.1038/s41598-024-57707-6 PMC10987622 38565594

[54] Zhu C , Xu Y , Jiang Z , et al. Disrupted hypothalamic CRH neuron responsiveness contributes to diet‐induced obesity. EMBO Rep. 2020;21(7):e49210. 32462726 10.15252/embr.201949210 PMC7332802

[55] Caruso A , Gaetano A , Scaccianoce S . Corticotropin‐Releasing Hormone: Biology and Therapeutic Opportunities. Biol‐Basel. 2022;11(12):1785. 10.3390/biology11121785 PMC9775501 36552294

[56] Kalsbeek A , Palm IF , La Fleur SE , et al. SCN outputs and the hypothalamic balance of life. J Biol Rhythms. 2006;21(6):458‐469. 17107936 10.1177/0748730406293854

[57] Li JD , Hu WP , Zhou QY . The circadian output signals from the suprachiasmatic nuclei. Prog Brain Res. 2012;199:119‐127. 22877662 10.1016/B978-0-444-59427-3.00028-9

[58] Campos LM , Cruz‐Rizzolo RJ , Watanabe IS , Pinato L , Nogueira MI . Efferent projections of the suprachiasmatic nucleus based on the distribution of vasoactive intestinal peptide (VIP) and arginine vasopressin (AVP) immunoreactive fibers in the hypothalamus of *Sapajus apella* . J Chem Neuroanat. 2014;57–58:42‐53. 10.1016/j.jchemneu.2014.03.004 24727411

[59] Ono D , Honma KI , Honma S . Roles of neuropeptides, VIP and AVP, in the mammalian central circadian clock. Front Neurosci. 2021;15:650154. 33935635 10.3389/fnins.2021.650154 PMC8081951

[60] Li JD , Burton KJ , Zhang C , Hu SB , Zhou QY . Vasopressin receptor V1a regulates circadian rhythms of locomotor activity and expression of clock‐controlled genes in the suprachiasmatic nuclei. Am J Physiol Regul Integr Comp Physiol. 2009;296(3):R824‐R830. 19052319 10.1152/ajpregu.90463.2008 PMC2665843

[61] Koshimizu TA , Nakamura K , Egashira N , Hiroyama M , Nonoguchi H , Tanoue A . Vasopressin V1a and V1b receptors: from molecules to physiological systems. Physiol Rev. 2012;92(4):1813‐1864. 23073632 10.1152/physrev.00035.2011

[62] Biag J , Huang Y , Gou L , et al. Cyto‐ and chemoarchitecture of the hypothalamic paraventricular nucleus in the C57BL/6J male mouse: a study of immunostaining and multiple fluorescent tract tracing. J Comp Neurol. 2012;520(1):6‐33. 21674499 10.1002/cne.22698 PMC4104804

[63] Chen J , Gomez‐Sanchez CE , Penman A , May PJ , Gomez‐Sanchez E . Expression of mineralocorticoid and glucocorticoid receptors in preautonomic neurons of the rat paraventricular nucleus. Am J Physiol Regul Integr Comp Physiol. 2014;306(5):R328‐R340. 24381176 10.1152/ajpregu.00506.2013 PMC3949076

[64] Leon‐Mercado L , Herrera Moro Chao D , Basualdo MD , Kawata M , Escobar C , Buijs RM . The arcuate nucleus: a site of fast negative feedback for corticosterone secretion in male rats. eNeuro. 2017;4(1):ENEURO.0350‐16.2017. 10.1523/ENEURO.0350-16.2017 PMC5334455 28275717

[65] Kwak SP , Young EA , Morano I , Watson SJ , Akil H . Diurnal corticotropin‐releasing hormone mRNA variation in the hypothalamus exhibits a rhythm distinct from that of plasma corticosterone. Neuroendocrinology. 1992;55(1):74‐83. 1319007 10.1159/000126099

[66] Girotti M , Weinberg MS , Spencer RL . Diurnal expression of functional and clock‐related genes throughout the rat HPA axis: system‐wide shifts in response to a restricted feeding schedule. Am J Physiol Endocrinol Metab. 2009;296(4):E888‐E897. 19190255 10.1152/ajpendo.90946.2008 PMC2670633

[67] Rohr KE , Telega A , Savaglio A , Evans JA . Vasopressin regulates daily rhythms and circadian clock circuits in a manner influenced by sex. Horm Behav. 2021;127:104888. 33202247 10.1016/j.yhbeh.2020.104888 PMC7855892

[68] Yamaguchi Y . Arginine vasopressin: Critical regulator of circadian homeostasis. Peptides. 2024;177:171229. 38663583 10.1016/j.peptides.2024.171229

[69] Watts AG , Tanimura S , Sanchez‐Watts G . Corticotropin‐releasing hormone and arginine vasopressin gene transcription in the hypothalamic paraventricular nucleus of unstressed rats: daily rhythms and their interactions with corticosterone. Endocrinology. 2004;145(2):529‐540. 14563696 10.1210/en.2003-0394

[70] Wunderer F , Kühne S , Jilg A , et al. Clock gene expression in the human pituitary gland. Endocrinology. 2013;154(6):2046‐2057. 23584858 10.1210/en.2012-2274

[71] Ikegami K , Nakajima M , Minami Y , Nagano M , Masubuchi S , Shigeyoshi Y . cAMP response element induces Per1 in vivo. Biochem Biophys Res Commun. 2020;531(4):515‐521. 32807491 10.1016/j.bbrc.2020.07.105

[72] Russell AL , Miller L , Yi H , Keil R , Handa RJ , Wu TJ . Knockout of the circadian gene, Per2, disrupts corticosterone secretion and results in depressive‐like behaviors and deficits in startle responses. BMC Neurosci. 2021;22(1):5. 33509094 10.1186/s12868-020-00607-y PMC7841886

[73] Kim ER , Xu Y , Cassidy RM , et al. Paraventricular hypothalamus mediates diurnal rhythm of metabolism. Nat Commun. 2020;11(1):3794. 32732906 10.1038/s41467-020-17578-7 PMC7393104

[74] Van Drunen R , Dai Y , Wei H , et al. Cell‐specific regulation of the circadian clock by BMAL1 in the paraventricular nucleus: Implications for regulation of systemic biological rhythms. Cell Rep. 2024;43(7):114380. 38935503 10.1016/j.celrep.2024.114380 PMC11446153

[75] Nakata M , Kumari P , Kita R , et al. Circadian Clock Component BMAL1 in the Paraventricular Nucleus Regulates Glucose Metabolism. Nutrients. 2021;13(12):4487. 34960038 10.3390/nu13124487 PMC8707417

[76] Perez‐Castro C , Renner U , Haedo MR , Stalla GK , Arzt E . Cellular and molecular specificity of pituitary gland physiology. Physiol Rev. 2012;92(1):1‐38. 22298650 10.1152/physrev.00003.2011

[77] Jiang W , Zhang H , Imachi H , et al. Corticotropin‐releasing hormone stimulates proopiomelanocortin transcription via the CaMKK/CaMKIV pathway in the AtT20 cell line. Cureus. 2025;17(4):e83257. 40453274 10.7759/cureus.83257 PMC12124421

[78] Kiessling S , Sollars PJ , Pickard GE . Light stimulates the mouse adrenal through a retinohypothalamic pathway independent of an effect on the clock in the suprachiasmatic nucleus. PLoS One. 2014;9(3):e92959. 24658072 10.1371/journal.pone.0092959 PMC3962469

[79] Son GH , Chung S , Choe HK , et al. Adrenal peripheral clock controls the autonomous circadian rhythm of glucocorticoid by causing rhythmic steroid production. Proc Natl Acad Sci U S A. 2008;105(52):20970‐20975. 19091946 10.1073/pnas.0806962106 PMC2634940

[80] Kiessling S , Eichele G , Oster H . Adrenal glucocorticoids have a key role in circadian resynchronization in a mouse model of jet lag. J Clin Invest. 2010;120(7):2600‐2609. 20577050 10.1172/JCI41192 PMC2898589

[81] Fahrenkrug J , Georg B , Hannibal J , Jørgensen HL . Altered rhythm of adrenal clock genes, StAR and serum corticosterone in VIP receptor 2‐deficient mice. J Mol Neurosci. 2012;48(3):584‐596. 22622901 10.1007/s12031-012-9804-7

[82] Engeland WC , Massman L , Mishra S , et al. The adrenal clock prevents aberrant light‐induced alterations in circadian glucocorticoid rhythms. Endocrinology. 2018;159(12):3950‐3964. 30321360 10.1210/en.2018-00769 PMC6240903

[83] Oster H , Damerow S , Kiessling S , et al. The circadian rhythm of glucocorticoids is regulated by a gating mechanism residing in the adrenal cortical clock. Cell Metab. 2006;4(2):163‐173. 16890544 10.1016/j.cmet.2006.07.002

[84] Fahrenkrug J , Hannibal J , Georg B . Diurnal rhythmicity of the canonical clock genes Per1, Per2 and Bmal1 in the rat adrenal gland is unaltered after hypophysectomy. J Neuroendocrinol. 2008;20(3):323‐329. 18208549 10.1111/j.1365-2826.2008.01651.x

[85] Sládek M , Lužná V , Houdek P , Sumová A . Suprachiasmatic nuclei possess glucocorticoid receptors that activate downstream signaling pathways but do not entrain their circadian clock. Acta Physiol (Oxf). 2026;242(1):e70138. 41314247 10.1111/apha.70138 PMC12662659

[86] Chung S , Son GH , Kim K . Circadian rhythm of adrenal glucocorticoid: its regulation and clinical implications. Biochim Biophys Acta. 2011;1812(5):581‐591. 21320597 10.1016/j.bbadis.2011.02.003

[87] Leliavski A , Dumbell R , Ott V , Oster H . Adrenal clocks and the role of adrenal hormones in the regulation of circadian physiology. J Biol Rhythms. 2015;30(1):20‐34. 25367898 10.1177/0748730414553971

[88] Husse J , Eichele G , Oster H . Synchronization of the mammalian circadian timing system: Light can control peripheral clocks independently of the SCN clock: alternate routes of entrainment optimize the alignment of the body's circadian clock network with external time. Bioessays. 2015;37(10):1119‐1128. 26252253 10.1002/bies.201500026 PMC5054915

[89] Zhang S , Dai M , Wang X , et al. Signalling entrains the peripheral circadian clock. Cell Signal. 2020;69:109433. 31982551 10.1016/j.cellsig.2019.109433

[90] Bechtel W . Hierarchy or heterarchy of mammalian circadian timekeepers? J Biol Rhythms. 2024;39(6):513‐534. 39449278 10.1177/07487304241286573 PMC11613639

[91] Bautista J , Ojeda‐Mosquera S , Ordóñez‐Lozada D , López‐Cortés A . Peripheral clocks and systemic zeitgeber interactions: from molecular mechanisms to circadian precision medicine. Front Endocrinol (Lausanne). 2025;16:1606242. 40510487 10.3389/fendo.2025.1606242 PMC12158691

[92] Inokawa H , Umemura Y , Shimba A , et al. Chronic circadian misalignment accelerates immune senescence and abbreviates lifespan in mice. Sci Rep. 2020;10(1):2569. 32054990 10.1038/s41598-020-59541-y PMC7018741

[93] Woller A , Gonze D . Circadian misalignment and metabolic disorders: a story of twisted clocks. Biol‐Basel. 2021;10(3):207. 10.3390/biology10030207 PMC8001388 33801795

[94] Buckley TM , Schatzberg AF . On the interactions of the hypothalamic‐pituitary‐adrenal (HPA) axis and sleep: normal HPA axis activity and circadian rhythm, exemplary sleep disorders. J Clin Endocrinol Metab. 2005;90(5):3106‐3114. 15728214 10.1210/jc.2004-1056

[95] Bec L , Herber R , Bailly S , et al. Sleep quality in glaucoma patients. Sci Rep. 2024;14(1):25593. 39462008 10.1038/s41598-024-76368-z PMC11513007

[96] Boivin DB , Boudreau P , Kosmadopoulos A . Disturbance of the circadian system in shift work and its health impact. J Biol Rhythms. 2022;37(1):3‐28. 34969316 10.1177/07487304211064218 PMC8832572

[97] Brum MCB , Senger MB , Schnorr CC , Ehlert LR , Rodrigues TDC . Effect of night‐shift work on cortisol circadian rhythm and melatonin levels. Sleep Sci. 2022;15(2):143‐148. 10.5935/1984-0063.20220034 PMC9210564 35755906

[98] Andreadi A , Andreadi S , Todaro F , et al. Modified cortisol circadian rhythm: the hidden toll of night‐shift work. Int J Mol Sci. 2025;26(5):2090. 40076739 10.3390/ijms26052090 PMC11899833

[99] Gubin D , Neroev V , Malishevskaya T , et al. Melatonin mitigates disrupted circadian rhythms, lowers intraocular pressure, and improves retinal ganglion cells function in glaucoma. J Pineal Res. 2021;70(4):e12730. 33730443 10.1111/jpi.12730

