# Supplementary material for: Retinal ganglion cell degeneration in glaucoma disrupts HPA axis temporal organization and dampens corticosterone production
Source: J Neuroendocrinol. 2026 Apr 24;38:e70182. doi: 10.1111/jne.70182 (PMC13108550; doi:10.1111/jne.70182)
Supplement: Supplementary file 1 — Table S1. Two‐way analysis of gene expression in the paraventricular nucleus. Table S2. Two‐way analysis of gene expression in the pituitary gland. Table S3. Rhythmic parameters of gene expression in the pituitary gland. Table S4. Two‐way analysis of gene expression in the adrenal gland. Table S5. Rhythmic parameters of gene expression in the adrenal gland. [file JNE-38-e70182-s001.docx]

**Table S1 – Two-way analysis of gene expression in the paraventricular nucleus**

**Table S2 – Two-way analysis of gene expression in the pituitary gland**

**Table S3 – Rhythmic parameters of gene expression in the pituitary gland**

**Table S4 – Two-way analysis of gene expression in the adrenal gland**

**Table S5 – Rhythmic parameters of gene expression in the adrenal gland**
